# Changes in highly sensitive alpha-fetoprotein for the prediction of the outcome in patients with hepatocellular carcinoma after hepatectomy

**DOI:** 10.1002/cam4.218

**Published:** 2014-03-03

**Authors:** Hidenori Toyoda, Takashi Kumada, Toshifumi Tada, Takanori Ito, Atsuyuki Maeda, Yuji Kaneoka, Chiaki Kagebayashi, Shinji Satomura

**Affiliations:** 1 Department of Gastroenterology, Ogaki Municipal HospitalOgaki, Japan; 2 Department of Surgery, Ogaki Municipal HospitalOgaki, Japan; 3 Wako Life Science Inc.Mountain View, California

**Keywords:** Changes, hepatectomy, hepatocellular carcinoma, highly sensitive measurement of AFP-L3, prognosis, tumor markers

## Abstract

We investigated changes in highly sensitive *lens culinaris* agglutinin A-reactive fraction of alpha-fetoprotein (hsAFP-L3) measured using a novel method and its predictive ability for prognosis in patients with hepatocellular carcinoma (HCC) who underwent curative hepatectomy, comparing to other HCC tumor markers, that is, AFP, des-gamma-carboxy prothrombin (DCP), and AFP-L3 measured with conventional method (cAFP-L3). AFP, DCP, and AFP-L3 including both cAFP-L3 and hsAFP-L3 were measured before and after curative hepatectomy in 187 patients. The percentage of patients with elevated tumor marker levels pre- and postoperatively was compared, and recurrence-free and overall survival rates were analyzed based on changes in tumor markers. The percentages of patients with elevated AFP, DCP, and cAFP-L3 decreased postoperatively. In contrast, the percentage of patients with elevated hsAFP-L3 did not decrease postoperatively. Both recurrence-free and overall survival rates were significantly lower in patients whose tumor marker levels remained elevated postoperatively than patients without tumor marker elevation postoperatively. Recurrence-free and overall survival rates of patients in whom hsAFP-L3 became elevated postoperatively despite normal preoperative hsAFP-L3 levels were significantly lower than those of patients with normal hsAFP-L3 postoperatively, and were similar to those of patients with persistent elevation. Preoperative elevations of AFP, DCP, and cAFP normalized in many patients postoperatively, but not for hsAFP-L3. The elevation of hsAFP-L3 identifies patients with poor prognosis despite the normalization of AFP and DCP.

## Introduction

Hepatocellular carcinoma (HCC) is one of the most common cancers in the world, and is the third most common cause of cancer-related death 1. Hepatectomy is usually a curative treatment for HCC with better prognosis than other treatment modalities including percutaneous locoregional therapies, transcatheter arterial chemoembolization, or sorafenib intake. However, as the outcome of patients treated with hepatectomy varies despite its curative intent, it is important to predict the outcome of patients with HCC who undergo hepatectomy.

Three tumor markers specific for HCC are currently used in several countries clinically: alpha-fetoprotein (AFP), *Lens culinaris* agglutinin A-reactive fraction of AFP (AFP-L3), and des-gamma-carboxy prothrombin (DCP), which is also known as protein induced by vitamin K absence/antagonist-II (PIVKA-II). The clinical utility of these tumor markers for the detection and diagnosis of HCC, evaluation of tumor progression, and determination of prognosis has been reported 2–5. Elevations in these tumor markers reflect the progression of HCC based on both imaging 6 and pathological examination 7. In addition to these functions, monitoring of changes in tumor markers with treatment is reportedly useful for the evaluation of treatment response 8–15. Decreases in and normalization of tumor markers are observed with several treatments for HCC including hepatectomy, locoregional therapy, transarterial chemoembolization, and systemic chemotherapy. Along with transplantation, hepatectomy is one of the treatment modalities for HCC with the highest curativity. Normalizations of tumor markers for HCC, therefore, are expected in many patients after hepatectomy 13,15. However, they sometimes remain elevated even after successful hepatectomy.

The changes in tumor markers with treatment and their association of outcomes were not clearly recognized in patients who underwent curative hepatectomy. In this study, we analyzed changes in HCC tumor markers after hepatectomy with curative intent and the significance of tumor marker treatment responses on patient outcomes. Especially, we measured AFP-L3 with two different methods, conventional method (cAFP-L3) and a new sensitive method (highly sensitive AFP-L3, hsAFP-L3), which showed improved utility in the diagnosis and the prediction of outcomes in patients with HCC 16, and evaluated changes in these two AFP-L3s after hepatectomy along with their ability to predict outcomes.

## Methods

### Patients

Between January 2004 and December 2011, 667 patients were diagnosed with primary, nonrecurrent HCC at our institution, of whom 288 were treated with hepatectomy. Stored serum samples were available for measuring the levels of three tumor markers, AFP, DCP, and AFP-L3 (conventional and highly sensitive), before and after hepatectomy in 187 patients. Decisions regarding each patient's treatment plan were based on the Japanese treatment guidelines for HCC 17. Anatomical hepatectomy was performed in all 187 patients. In all patients, HCC tumors were resected with ample margins and enucleation without adequate margins was not performed. The diagnosis of HCC was confirmed by pathologic examination of resected specimens and the absence of HCC tumor cells on the margin of the resected specimen was confirmed pathologically.

One month after hepatectomy, all patients underwent computed tomography (CT) examination of the thorax, and the abdomen to confirm the absence of residual HCC. All patients were followed up for a median of 41.9 months (range, 3.1–137.9 months) until death or December 2012, whichever came first, at our institution, with ultrasound (US) and additional CT or magnetic resonance imaging (MRI), every 3–6 months. Regular monitoring of tumor markers was performed every 3 months. If an elevation of in one or more tumor markers was detected, additional imaging tests (usually CT or MRI) were performed to check for recurrence. If recurrence was confirmed, patients underwent treatment for recurrent HCC based on treatment guidelines.

The study protocol was approved by the institutional review board and was in compliance with the Declaration of Helsinki.

### Assays of AFP, DCP, and AFP-L3

Pretreatment tumor markers were measured within 1 week of hepatectomy. Posttreatment tumor markers were measured in the serum sample obtained during the first patient visit between 1 and 2 months after hepatectomy. The reported half-life of AFP and AFP-L3 is 4 days 18 and the half-life of DCP is 60 h 19. Therefore, the values of posttreatment tumor markers were not influenced by pretreatment tumor marker elevations. Serum AFP levels were determined using an enzyme-linked immunosorbent assay in a commercially available kit (ELISA-AFP, International Reagents, Kobe, Japan). Serum DCP levels were determined using a sensitive enzyme immunoassay (Eitest PIVKA-II kit, Eisai Laboratory, Tokyo, Japan) according to the manufacturer's instructions 20–22. Conventional measurement of AFP-L3 was performed using a column chromatography and liquid-phase binding assay on a LiBASys autoanalyzer (Wako Pure Chemical Industries, Ltd., Osaka, Japan) 23,24. Highly sensitive measurement of AFP-L3 was achieved using a microchip capillary electrophoresis and liquid-phase binding assay on a *μ*TASWako i30 autoanalyzer (Wako Pure Chemical Industries, Ltd.) 25. The cut-off value of 20 ng/mL was used to establish positivity for AFP, as proposed by Oka et al. and Koda et al. 26,27. The cut-off value used to establish positivity for DCP was 40 mAU/mL, as proposed by Okuda et al. 28. The cut-off value used to establish positivity for conventional AFP-L3 was 10%, as proposed by Shimizu et al. 29. The cut-off value used to establish positivity for hsAFP-L3 was 5% based on our previous study 30.

### Statistical analyses

Differences in percentages between groups were analyzed using the chi-square test. Differences in mean quantitative values were analyzed using the Mann–Whitney *U* test. The date of hepatectomy was defined as time zero for calculating survival rate. In the analysis of survival rates, patients who died were noncensored and surviving patients were censored. When recurrence-free survival rates and overall survival rates were compared based on the changes in tumor markers after hepatectomy, patients were categorized into group A when tumor marker levels were normal both before and after hepatectomy. Patients were categorized into group B when tumor marker levels were elevated before hepatectomy but normalized after hepatectomy. Patients were categorized into group C when tumor marker levels were elevated both before and after hepatectomy. Patients were categorized into group D when tumor marker levels were normal before hepatectomy but elevated after hepatectomy. The Kaplan–Meier method 31 was used to calculate survival rates, and the log-rank test 32 was used to analyze differences in survival. Data analyses were performed using JMP statistical software, version 6.0 (Macintosh version; SAS Institute, Cary, NC). All *P* values were derived from two-tailed tests, with *P* < 0.05 considered to indicate statistical significance.

## Results

### Clinical features of patients and HCC

Table 1 summarizes the pretreatment characteristics of the study patients. This population was comprised of 140 males and 47 females with a mean age of 67.2 ± 8.7 years. Most (95.7%) patients belonged to Child-Pugh class 33 A. Multiple tumors were present in 18.2% of patients. Portal vein invasion was observed in 3.2% of patients based on pretreatment imaging studies. Pretreatment AFP, DCP, cAFP-L3, and hsAFP-L3 were above the specified cut-off levels in 35.8%, 50.3%, 19.8%, and 45.5% of patients, respectively.

**Table 1:** Characteristics of study patients (*n* = 187)

Age, years (range)	67.2 ± 8.7 (21–83)
Sex (female/male)	47 (25.1)/140 (74.9)
Etiology (HBV/HCV/HBV + HCV/non-HBV, non-HCV)	31 (16.6)/123 (65.8)/2 (1.0)/31 (16.6)
Child-Pugh class (A/B)	179 (95.7)/8 (4.3)
Albumin (g/dL)	4.04 ± 0.42
Total bilirubin (mg/dL)	0.78 ± 0.33
ICG retention rate at 15-min (%)	15.4 ± 7.4
Prothrombin (%)	92.6 ± 14.1
Platelet (×1000/mL)	145 ± 70
Tumor size, cm (range)	3.24 ± 2.52 (0.8–16.4)
Number of tumors, *n* (range)	1.27 ± 0.63 (1–4)
(single/multiple)	153 (81.8)/34 (18.2)
Macroscopic portal vein invasion (absent/present)^1^	181 (96.8)/6 (3.2)
AFP (ng/mL); median (range)	11.1 (0.8–27,242.8)
≥20/<20 ng/mL	67 (35.8)/120 (64.2)
DCP (mAU/mL); median (range)	39.0 (5.0–60,030.0)
≥40/<40 mAU/mL	94 (50.3)/93 (49.7)
Conventional AFP-L3 (%); median (range)	0.5 (0.0–87.2)
≥10/<10%	37 (19.8)/150 (80.2)
Highly sensitive AFP-L3 (%); median (range)	4.8 (0.0–89.7)
≥5/<5%	85 (45.5)/102 (54.5)

Values are means ± SD, unless otherwise indicated. Percentages are given in parentheses, unless otherwise indicated. HBV, hepatitis B virus; HCV, hepatitis C virus; ICG, indocyanine green test; AFP, alpha-fetoprotein; AFP-L3, *Lens culinaris* agglutinin-reactive AFP; DCP, des-gamma-carboxy prothrombin.

1 Evaluated based on imaging findings.

### Changes in HCC tumor markers with hepatectomy

**Figure 1:**
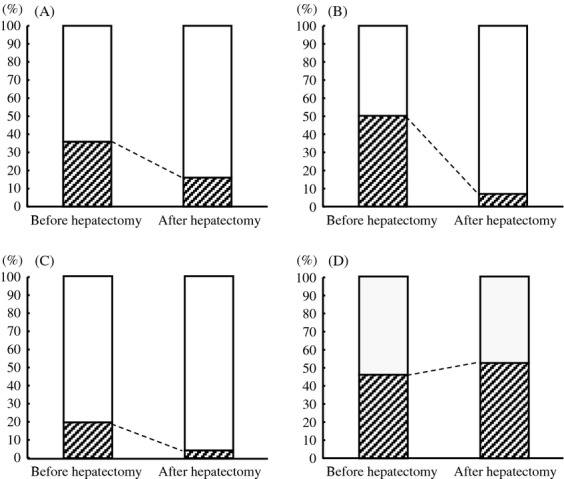
Changes in the percentages of patients with elevated tumor markers before and after hepatectomy (*n* = 187). (A) Percentage of patients with AFP ≥ 20 ng/mL. The percentage decreased significantly after hepatectomy (35.8% before hepatectomy vs. 16.6% after hepatectomy, *P* < 0.0001). (B) Percentage of patients with DCP ≥ 40 mAU/mL. The percentage decreased significantly after hepatectomy (50.3% before hepatectomy vs. 7.0% after hepatectomy, *P* < 0.0001). (C) Percentage of patients with conventional AFP-L3 ≥ 10%. The percentage decreased significantly after hepatectomy (19.8% before hepatectomy vs. 7.0% after hepatectomy, *P* = 0.0005). (D) Percentage of patients with highly sensitive AFP-L3 ≥ 5%. The percentage was similar before and after hepatectomy (45.5% before hepatectomy vs. 52.4% after hepatectomy, *P* = 0.2145).

**Figure 2:**
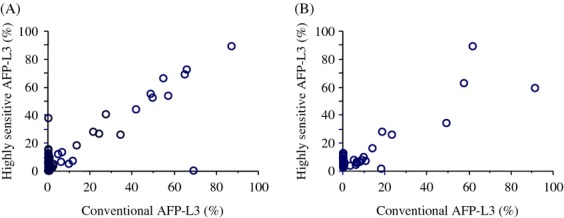
Correlation between conventional AFP-L3 and highly sensitive AFP-L3 levels (A) before and (B) after hepatectomy. The correlation of AFP-L3 measured with two different methods decreased after hepatectomy (r^2^, 0.76 before hepatectomy and 0.47 after hepatectomy).

Figure 1 compares the changes in the percentage of patients with elevated tumor markers for HCC before and after hepatectomy. The percentage of patients with elevated AFP, DCP, and conventional AFP decreased with hepatectomy (AFP, 35.8% before hepatectomy vs. 16.6% after hepatectomy, *P* < 0.0001; DCP, 50.3% before hepatectomy vs. 7.0% after hepatectomy, *P* < 0.0001; cAFP-L3, 19.8% before hepatectomy vs. 7.0% after hepatectomy, *P* = 0.0005). In contrast, the percentage of patients with elevated hsAFP-L3 did not change with hepatectomy (45.5% before hepatectomy vs. 52.4% after hepatectomy, *P* = 0.2145). None of patients with normal AFP, DCP, and cAFP-L3, respectively, prior to hepatectomy had elevated levels after hepatectomy (group D). HsAFP-L3 was elevated after hepatectomy in 34 of 101 patients (33.7%) whose levels were normal before hepatectomy (group D), whereas 22 of 86 patients (25.6%) with elevated hsAFP-L3 levels before hepatectomy had normalized postoperative values (group B). Figure 2 shows the correlation between cAFP-L3 and hsAFP-L3 before (A) and after (B) hepatectomy. The correlation of AFP-L3 measured with two different methods decreased after hepatectomy (*r*^2^, 0.76 before hepatectomy and 0.47 after hepatectomy).

### Recurrence-free and overall survival rates of patients after hepatectomy based on pretreatment elevations of tumor markers and changes after hepatectomy

**Figure 3:**
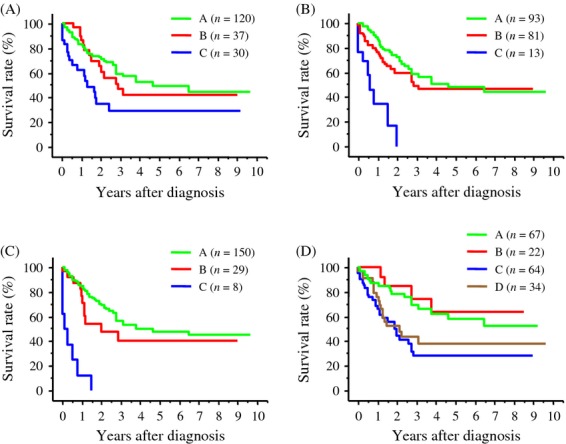
Recurrence-free survival rates of patients stratified based on the treatment response of tumor markers after hepatectomy. A: Tumor marker was normal before and after hepatectomy. B: Tumor marker was elevated before hepatectomy but normalized after hepatectomy. C: Tumor marker was elevated before and after hepatectomy. D: Tumor marker was normal before hepatectomy but elevated after hepatectomy. (A) Survival rates based on the treatment response of AFP. The survival rate of patients in group C was significantly lower than that of patients in group A (*P* = 0.0016) and in group B (*P* = 0.0345). (B) Survival rates based on the treatment response of DCP. The survival rate of patients in group C was significantly lower than that of patients in group A (*P* < 0.0001) and in group B (*P* = 0.0003). (C) Survival rates based on the treatment response of conventional AFP-L3. The survival rate of patients in group C was significantly lower than that of patients in groups A and B (both, *P* < 0.0001). (D) Survival rates based on the treatment response of highly sensitive AFP-L3. The survival rate of patients in group C was significantly lower than that of patients in group A (*P* = 0.0005) and patients in group B (*P* = 0.0065). The survival rate of patients in group D was significantly lower than that of patients in group A (*P* = 0.0024) and patients in group B (*P* = 0.0345).

**Figure 4:**
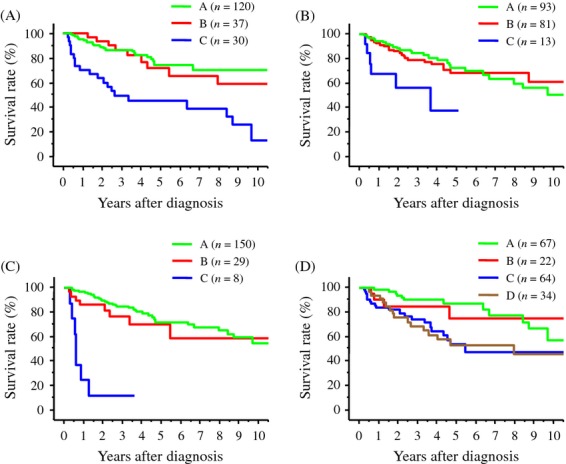
Overall survival rates of patients stratified based on the treatment response of tumor markers after hepatectomy. A: Tumor marker was normal before and after hepatectomy. B: Tumor marker was elevated before hepatectomy but normalized after hepatectomy. C: Tumor marker was elevated before and after hepatectomy. D: Tumor marker was normal before hepatectomy but elevated after hepatectomy. (A) Survival rates based on the treatment response of AFP. The survival rate of patients in group C was significantly lower than that of patients in group A (*P* < 0.0001) and in group B (*P* = 0.0012). (B) Survival rates based on the treatment response of DCP. The survival rate of patients in group C was significantly lower than that of patients in group A (*P* = 0.0016) and in group B (*P* = 0.0091). (C) Survival rates based on the treatment response of conventional AFP-L3. The survival rate of patients in group C was significantly lower than that of patients in groups A and B (both, *P* < 0.0001). (D) Survival rates based on the treatment response of highly sensitive AFP-L3. The survival rate of patients in group C was significantly lower than that of patients in group A (*P* = 0.0026). The survival rate of patients in group D was significantly lower than that of patients in group A (*P* = 0.0024).

Recurrence-free and overall survival rates were compared based on pretreatment elevations of tumor markers and changes after hepatectomy (Fig. 3, 4). Both recurrence-free and overall survival rates were significantly lower in patients with persistent elevations of AFP, DCP, and cAFP-L3, respectively, before and after hepatectomy (group C) than in both patients without elevation of tumor markers (preoperatively and postoperatively, group A) and patients whose pretreatment tumor marker levels elevated but normalized after hepatectomy (group B). Recurrence-free and overall survival rates were significantly lower in patients with persistently elevated levels of hsAFP-L3 before and after hepatectomy (group C) than patients with normal levels of hsAFP-L3 before and after hepatectomy (group A). Recurrence-free survival rate was significantly lower in patients with persistently elevated levels of hsAFP-L3 before and after hepatectomy (group C) than patients whose pretreatment tumor marker levels elevated but normalized after hepatectomy (group B). In contrast, both recurrence-free and overall survival rates of patients with normal hsAFP-L3 levels before hepatectomy but elevated levels after hepatectomy (group D) was significantly lower than those of patients with normal postoperative levels of hsAFP-L3 regardless of pretreatment levels (groups A and B), and the rate was similar to that of patients with persistent elevations of hsAFP-L3 before and after hepatectomy (group C).

### HCC characteristics based on the elevations of conventional and highly sensitive AFP-L3 after hepatectomy

Table 2 lists the characteristics of HCC tumors according to elevations of conventional and hsAFP-L3 after hepatectomy. The size of HCC was significantly greater in patients with elevations in cAFP-L3 than in patients without the elevation (*P* = 0.0143). The percentages of moderately or poorly differentiated HCC and HCC with infiltrative growth was significantly higher in patients with elevations in cAFP-L3 than in patients without the elevation (*P* = 0.0454 and *P* = 0.0203, respectively). In contrast, no differences were found in the characteristics of HCC between patients with and without postoperative elevations of hsAFP-L3. Although postoperative total AFP was elevated (≥20 ng/mL) in 10 of 13 patients (76.9%) in whom cAFP-L3 was elevated postoperatively, total AFP was elevated in only 22 of 98 patients (22.4%) in whom postoperative elevations of hsAFP-L3 were observed. Especially, the total AFP concentration after hepatectomy was within normal range (<20 ng/mL) in all patients in whom hsAFP-L3 became elevated after hepatectomy despite the normal values before hepatectomy (data not shown).

**Table 2:** Characteristics of resected hepatocellular carcinoma specimens according to elevations of conventional or highly sensitive AFP-L3 after hepatectomy (*n* = 187)

	Conventional AFP-L3		Highly sensitive AFP-L3	
		
	Negative (*n* = 174)	Positive (*n* = 13)	Negative (*n* = 89)	Positive (*n* = 98)
Child-Pugh class (A/B)	166 (95.4)/8 (4.6)	13 (100)/0	87 (97.8)/2 (2.2)	92 (93.9)/6 (6.1)
Tumor size (cm)	3.09 ± 2.30^a^	5.19 ± 4.21^a^	3.28 ± 2.56	3.20 ± 2.50
Number of tumors (single/multiple)	143 (82.2)/31 (17.8)	10 (76.9)/3 (23.1)	73 (82.0)/16 (18.0)	80 (81.6)/18 (18.4)
Differentiation (well-/moderately or poorly)	52 (29.9)/122 (70.1)^2^	0/13 (100)^b^	25 (29.1)/64 (71.9)	27 (27.6)/71 (72.4)
Growth pattern (expansive/infiltrative)	161 (92.5)/13 (7.5)^c^	9 (69.2)/4 (30.8)^c^	81 (91.0)/8 (9.0)	89 (90.8)/9 (9.2)
Capsular formation (absent/present)^1^	62 (38.5)/99 (61.5)	2 (22.2)/7 (77.8)	28 (34.6)/53 (65.4)	36 (40.4)/53 (59.6)
Capsular infiltration (absent/present)^2^	38 (38.4)/61 (61.6)	1 (14.3)/6 (85.7)	23 (43.4)/30 (56.6)	16 (30.2)/37 (69.8)
Portal vein invasion (absent/present)^3^	146 (83.9)/28 (16.1)	8 (61.5)/5 (38.5)	69 (77.5)/20 (22.5)	85 (86.7)/13 (13.3)

Unless otherwise indicated, values are means ± SD and percentages are indicated in parentheses. ^a^*P* = 0.0143, ^b^*P* = 0.0454, ^c^*P* = 0.0203.

1 Evaluated only in HCC with expansive growth.

2 Evaluated only in HCC with capsular formation.

3 On pathologic evaluation.

## Discussion

In this study, we investigated changes in newly developed hsAFP-L3 in patients treated with hepatectomy with curative intent. The analytical sensitivity of the conventional assay system for AFP-L3 is insufficient in patients with low total AFP levels; cAFP-L3 cannot be measured when total AFP is less than 10 ng/mL 23,24. The new generation of assays for AFP-L3 (micro total analysis system; *μ*TAS), which used novel advanced microfluidics-based separation technology, has enabled the accurate measurement of AFP-L3 even at very low total AFP concentrations 16,25,30,34–37. With this method, the percentage of hsAFP-L3 is measurable when total AFP concentration is 2 ng/mL or higher.

The percentage of patient with elevated AFP, DCP, and cAFP-L3 decreased after hepatectomy with normalization of these values in patients with elevated pretreatment value. Surprisingly, in contrast, the percentage of patients with elevated hsAFP-L3 did not decrease after hepatectomy, despite the high curativity. The percentage of patients in whom hsAFP-L3 was elevated after hepatectomy was greater than 50% despite its curative intent. The percentage of AFP-L3 is calculated as the serum AFP-L3 concentration divided by the total serum AFP concentration. Therefore, the analytical sensitivity for AFP-L3 depends on the concentration of total AFP. The improved sensitivity of AFP-L3 measurement might, therefore, account for this unexpected change in the percentage of patients with elevated hsAFP-L3 after hepatectomy. The decrease in the correlation between cAFP-L3 and hsAFP-L3 after hepatectomy will be due to the elevation of hsAFP-L3 in patients with normal total AFP after hepatectomy. Indeed, the total AFP concentration after hepatectomy was within normal range (<20 ng/mL) in all patients in whom hsAFP-L3 became elevated after hepatectomy despite its normal range before hepatectomy.

The postoperative elevation of respective tumor markers predicts patient recurrence-free and overall survival after hepatectomy regarding to all tumor markers. Both recurrence-free and overall survival rates of patients in whom tumor markers were persistently elevated before and after hepatectomy was lower than those of patients with normal tumor marker levels after hepatectomy that included patients whose tumor markers were not elevated before and after hepatectomy and patients whose elevated pretreatment values normalized after hepatectomy, for AFP, DCP, and cAFP-L3. This was similar for hsAFP-L3. In addition, the survival rate of patients whose normal pretreatment hsAFP-L3 levels became elevated after hepatectomy was lower than that of patients with normal posthepatectomy hsAFP-L3 levels and was comparable to patients with persistently elevated hsAFP-L3 before and after hepatectomy, showing that postoperative elevation of hsAFP-L3 levels predict unfavorable outcome regardless of its pretreatment status.

It is unknown why hsAFP-L3 levels increase after hepatectomy despite normal preoperative levels in some patients. We did not find any differences in the characteristics of HCC between patients with and without elevations of hsAFP-L3 after hepatectomy, whereas HCC had more advanced characteristics in patients with postoperative elevations of cAFP-L3. The elevation of cAFP-L3 after hepatectomy might partly be explained by potential residual HCC cells in the liver that were not detected by imaging studies postoperatively, due to the progressive nature of resected HCC. In contrast, postoperative elevation of hsAFP-L3 was not associated with morphologic and pathologic findings of resected HCC. Postoperative elevations of hsAFP-L3, therefore, cannot be predicted based on the progression of resected HCC. The postoperative elevation of hsAFP-L3 may reflect undefined malignant potential of HCC tumor that was not shown by pathologic and imaging examinations.

In conclusion, this study showed that the percentage of patients with elevated hsAFP-L3 did not decrease after hepatectomy when it was measured with the highly sensitive method, although the percentage of patients with elevated AFP, DCP, and cAFP-L3 levels decreased markedly. Postoperative elevations of hsAFP-L3 were observed in more than half of the patients who underwent hepatectomy with curative intent, and suggest unfavorable outcome.
